# A genetic and developmental biological approach for a family with complex congenital heart diseases—evidence of digenic inheritance

**DOI:** 10.3389/fcvm.2023.1135141

**Published:** 2023-04-25

**Authors:** Yu Yoshida, Keiko Uchida, Kazuki Kodo, Reina Ishizaki-Asami, Jun Maeda, Yoshinori Katsumata, Shinsuke Yuasa, Keiichi Fukuda, Kenjiro Kosaki, Yusuke Watanabe, Osamu Nakagawa, Hiroyuki Yamagishi

**Affiliations:** ^1^ Department of Pediatrics, Keio University School of Medicine, Tokyo, Japan; ^2^ Health Center, Keio University, Kanagawa, Japan; ^3^ Department of Cardiology, Keio University School of Medicine, Tokyo, Japan; ^4^ Center for Medical Genetics, Keio University School of Medicine, Tokyo, Japan; ^5^ Department of Molecular Physiology, National Cerebral and Cardiovascular Center Research Institute, Osaka, Japan

**Keywords:** trio gene analysis, nodal, tbx20, complex congenital heart disease, rare variants

## Abstract

**Objective:**

Congenital heart disease (CHD) is caused by cardiovascular developmental defects and has a global prevalence of ∼1%. The etiology of CHD is multifactorial and remains generally unknown, despite advances in analytical techniques based on next-generation sequencing (NGS). The aim of our study was to elucidate the multi-genetic origin and pathogenesis of an intriguing familial case with complex CHD.

**Methods:**

We performed an original trio-based gene panel analysis using NGS of the family, including two siblings with CHD of single ventricular phenotype, and their unaffected parents. The pathogenicity of the detected rare variants was investigated *in silico*, and the functional effects of the variants were confirmed *in vitro* using luciferase assays. The combinatorial effect of gene alterations of the putative responsible genes was tested *in vivo* using genetically engineered mutant mice.

**Results:**

NGS-based gene panel analyses revealed two heterozygous rare variants in *NODAL* and in *TBX20* common to the siblings and to just one of parents. Both variants were suspected pathogenic *in silico,* and decreased transcriptional activities of downstream signaling pathways were observed *in vitro*. The analyses of *Nodal* and *Tbx20* double mutant mice demonstrated that *Nodal^+/−^Tbx20^−/−^* embryos showed more severe defects than *Nodal^+/+^Tbx20^−/−^* embryos during early heart development. The expression of *Pitx2*, a known downstream target of *Nodal*, was downregulated in *Tbx20^−/−^* mutants.

**Conclusions:**

Two rare variants on *NODAL* and *TBX20* genes detected in this family were considered to be loss-of-function mutations. Our results suggest that *NODAL* and *TBX20* may be complementary for the cardiac development, and a combinatorial loss-of-function of *NODAL* and *TBX20* could be implicated in digenic inherence as the etiology of complex CHD associated with single ventricle defects in this family.

## 1. Introduction

Congenital heart disease (CHD) is caused by defects in cardiovascular development during the first trimester of gestation and has an incidence of ∼2.5 to 13.8 per 1,000 births (1). The etiology of most CHDs remains generally unknown and is largely attributed to the so-called multifactorial, including genetic and environmental, factors. The genetic factors associated with CHD include chromosomal abnormalities in 12%, *de novo* copy number variants such as chromosomal microdeletion in 15%, *de novo* gene mutation affecting protein function in 10%, and inherited gene mutations in 1.3% of cases. About two to four hundred genes have been implicated in CHD, encompassing transcription factors, cell signaling and adhesion molecules, and structural proteins that are involved in heart development (1, 2). Scientific advances, such as next-generation sequencing (NGS), help identify pathogenic variants associated with CHD with Mendelian patterns (3). However, the lack of experimental approaches fails to clarify the involvement of multiple genetic variants, especially oligogenic or polygenic inheritance and genetic modifiers, that lead to complex phenotypes.

NGS-based gene panel testing, which can be complemented with array comparative genomic hybridization and other methods, provides a comprehensive, timely, and efficient approach for the molecular diagnosis of heterogeneous disorders, such as CHD. Compared to single-gene test and whole exome sequencing analyses, NGS-based gene panel analysis increases analytical sensitivity for genetic diagnostic testing and can save time and money. It also simplifies the decision-making evidence for physicians, because the likelihood of discovering multiple confounding variants of unknown clinical significance is less than that when using whole-exome sequencing (4).

In this study, we performed genetic analyses of a family with complex CHD. Through NGS-based gene panel testing, *in vitro* functional analyses of the variants of interest, and *in vivo* function analyses using genetically engineered animal models, we attempted to provide evidence of digenic inheritance in complex CHD.

## 2. Methods

### 2.1. Clinical evaluation of patients and DNA collection

The patients were diagnosed as having congenital cardiac anomalies before birth. They were hospitalized in Keio University Hospital at birth and made a definitive diagnosis based on their clinical history, physical examination, echocardiogram, and heart catheterization by well-trained pediatric cardiologists. We checked that their parents do not have any cardiac diseases through oral interview and physical examination.

The parents provided written informed consent for this research on them and their children. Blood samples were obtained after receiving written informed consent.

Genomic DNA (gDNA) was extracted directly from whole blood using the Gentra Puregene Blood Kit (Qiagen, Venlo, The Netherlands), according to the manufacturer's protocol.

### 2.2. NGS

Using NGS, the patients were screened for genomic variants in cardiac disease-specific genes. A total of 197 genes were selected and subjected to gene panel analysis using NGS conducted by the Center for Medical Genetics and the Department of Cardiology, Keio University School of Medicine (Supplementary Table S1).

### 2.3. Direct sequencing and variant analysis

Genetic variants detected using NGS were reconfirmed using Sanger sequencing. Arrays containing these variants in gDNA samples were amplified using polymerase chain reaction (PCR) and TaKaRa Ex Taq Hot Start Version (Takara Bio, Kusatsu, Japan). All reactions were performed using a DNA Engine Peltier Thermal Cycler (Bio-Rad Laboratories, Hercules, CA, USA). The PCR products puriﬁed using NucleoSpin Extract II Kit (Takara Bio) were used for direct bidirectional sequencing to search for nucleotide changes, using the BigDye Terminator v3.1 Cycle Sequencing Kit (Applied Biosystems, Waltham, MA, USA) and a 3130xl Genetic Analyzer (Applied Biosystems).

### 2.4. *In silico* analyses of detected sequence variants

The detected sequence variants were validated using the UCSC genome browser (https://genome-asia.ucsc.edu, human assembly December 2013, GRch38/hg38), Ensembl (http://www.ensembl.org, Release 105), and ClinVar (https://www.ncbi.nlm.nih.gov/clinvar/, updated in September 2021) database. SIFT (http://sift.jcvi.org, version 5.2.2) and PolyPhen-2 (http://genetics.bwh.harvard.edu/pph2, version 2.2.2) were used for analyzing the missense variants.

### 2.5. Plasmid construction and site-directed mutagenesis

A human cDNA clone of *NODAL* (ID; X01Y107A01) in pGEM-T Easy vector was purchased from DNAFORM (Yokohama, Japan), and a human cDNA clone of *TBX20* (ID; FXC03772) in pF1K vector was purchased from Kazusa Genome Technologies (Kisarazu, Japan). To construct the variant cDNA, site-directed mutagenesis was performed using the PrimeSTAR Max Mutagenesis Basal Kit (Takara Bio), according to the manufacturer's instructions. All the PCR products were verified using sequencing.

### 2.6. Western blot

The PCR products (both wildtype and mutated type) were subcloned into another mammalian cDNA expression vector (pCMV-Tag3; Agilent Technologies, Santa Clara, CA, USA). The c-Myc tag was used for the detection of the recombinant fusion protein. Proteins were prepared from HEK293 cells transfected with the expression vectors using Lipofectamine 2000. c-Myc-tagged NODAL or TBX20 proteins were detected by western blotting using monoclonal anti-c-Myc antibodies, goat anti-mouse IgG-HRP (Santa Cruz Biotechnology, Dallas, TX), and SuperSignal West Femto Maximum Sensitivity Substrate (Thermo Scientific, Waltham, MA) after SDS-polyacrylamide gel electrophoresis.

### 2.7. Luciferase assays

For analysis of NODAL function, P19 cells, a cell line isolated from the embryo of a male mouse with teratocarcinoma, were transfected using Lipofectamine 2000 (Thermo Fisher Scientific, Waltham, MA, USA) with 200 ng p(Smad binding element, SBE)_4_ luciferase reporter plasmid (Addgene, Cambridge, MA, USA) as a reporter vector, 125 ng of *NODAL* expression vector (Dnaform, Kanagawa, Japan), 42 ng each of *CRIPTO*, *CRYPTIC* and *GDF1,* expression vectors (5) as enhancers for *NODAL*, and 50 ng of pRL-SV40 Vector (Promega, Madison, WI, USA) as an internal control.

For analysis of TBX20 function, HeLa cells were transfected using Lipofectamine 2000 (Thermo Fisher Scientific) with 125 ng of *NPPA* promoter-luciferase plasmid as a reporter vector, 125 ng of *TBX20* expression vector, 125 ng each of *GATA4* and *NKX2.5* expression vectors as enhancers for *TBX20*, and 50 ng of pRL-SV40 Vector (Promega) as an internal control.

Luciferase activity was measured 42 h after transient transfection using the Dual-Luciferase Reporter Assay System (Promega) and Synergy4 (BioTek Instruments, Winooski, VT, USA) or Cytation5 (BioTek Instruments), according to the manufacturer's instructions. All experiments were repeated five times.

### 2.8. Mice, *in situ* hybridization, and quantitative Rt-PCR

*Nodal^+/−^* and *Tbx20^+/−^* mouse lines were used, as described previously (6, 7). *Nodal^+/−^Tbx20^−/−^* murine embryos were generated by intercrossing *Nodal^+/−^Tbx20^+/−^* and *Tbx20^+/−^* mice. Embryonic day (E) 8.75 to E9.5, mothers were euthanized using cervical dislocation, and their uteri were dissected to harvest embryos. PCR genotyping was performed using the following primers: forward, 5′-ataggcacgtgctctcccaa-3′ (for wildtype) or 5′-gagccatttggttctccctg-3′ (for mutant); reverse, 5′-ccccacacctcacataacct-3′ for the *Nodal* line, and forward, 5′-aataaatcggcccttgttct-3′; reverse, 5′-ggaggctttcgaaaagtgaa-3′ for the *Tbx20* line. For *Pitx2* expression analysis*,* whole-mount *in situ* hybridization using E9.5 embryos was performed as described previously (8, 9). Briefly, total RNA was extracted from E9.5 embryos, and cDNA was synthesized using High-Capacity RNA-to-cDNA kit (Thermo Fisher Scientific). Quantitative RT-PCR analysis was performed with Power SYBR Green PCR Master Mix and a StepOnePlus system (Thermo Fisher Scientific). Glyceraldehyde-3-phosphate dehydrogenase (*Gapdh*) was used as an internal control. Primer sequences used for *Pitx2c* and *Gapdh* are as follows: forward, 5′-ccctgaagtcgcagagaaaga-3′; reverse, 5′-agtgaaatgagtcctctgccg-3 ′, and forward, 5′-tgaaggtcggtgtgaacgg-3′; reverse, 5′-cgtgagtggagtcatactggaa-3′.

### 2.9. Statistical analyses

For luciferase assays, data are presented as normalized relative light units (fold activation). Data in luciferase assays and quantitative RT-PCRwere analyzed using one-way ANOVA followed by Tukey's *post hoc* test. Statistical significance was set at *p* < 0.05. R (version 4.2.2, https://www.r-project.org/) was used for statistical analysis.

### 2.10. Ethics approval

This study was conducted according to the principles of the Declaration of Helsinki (1989). Clinical evaluations were approved by the Internal Ethics Committee of Keio University School of Medicine (approval number 20120042) and were conducted only after written informed consent had been obtained. All animal protocols were approved by the Institutional Animal Care and Use Committees of Keio University [approval number 09122-(13), A2022-286] and of National Cerebral and Cardiovascular Center (approval number 19026 and 19-6), and conformed to the National Institutes of Health Guidelines for the Care and Use of Laboratory Animals. The mice were anesthetized by intraperitoneal injection of 0.75 mg/kg of medetomidine, 4.0 mg/kg of midazolam, and 5.0 mg/kg of butorphanol and then euthanized by cervical dislocation.

## 3. Results

### 3.1. A familial case with CHD suggests multifactorial inheritance

**Figure 1:**
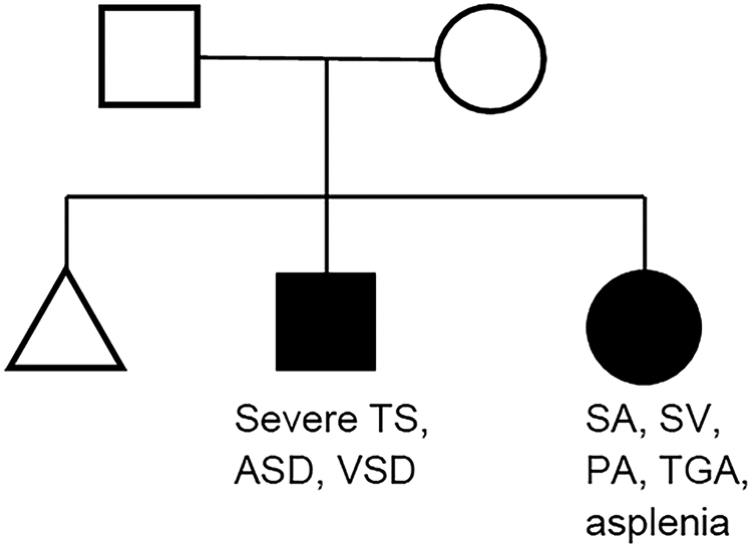
Pedigree chart of the family. TS, tricuspid valve stenosis; ASD, atrial septal defect; VSD, ventricular septal defect; SA, single atrium; SV, single ventricle; PA, pulmonary atresia; TGA, transposition of the great arteries.

The pedigree chart of our patients is shown in Figure 1. Neither parent had heart disease or family history of heart disease. Their first child was a miscarriage without phenotype or genotype analysis. Their second child, a male, has severe tricuspid stenosis, atrial septal defect, and ventricular septal defect, manifesting a functional left-sided single ventricle. Their third child, a female, has right-sided single ventricle with asplenia-type heterotaxy.

From the pedigree, an autosomal recessive inheritance was assumed for siblings with CHD at first. Cardiac phenotype seemed common regarding as the single ventricular morphology between siblings; however, their detailed phenotypes were different (single left ventricle with severe tricuspid stenosis vs. single right ventricle with heterotaxy).

### 3.2. Four sequence variants identified in the family

NGS using gene panels for gDNA from the siblings identified four common missense variants in four genes that were candidates for the genetic cause of CHD in the siblings. All these variants were confirmed by direct sequencing (Table 1). In the analyses of gDNA from the parents, a heterozygous variant in *NODAL* (c.1021G > T, p.V341l) common to the siblings was found in the father, whereas three heterozygous variants in *CASQ2* (c.1163A > G, p.E388G), *SYNE1* (c.17867G > A, p.R5956H), and *TBX20* (c.991A > G, p.T331A) common to the siblings were found in the mother.

**Table 1:** Detected variants from the siblings with CHDs.

gene	Nucleotide change	Amino acid change	Existence of the heterozygous variant				dbSNP	Allele frequency^a^ (%)	SIFT	PolyPhen-2
			brother	sister	father	mother				
NODAL	1021G > T	V341l	yes	yes	yes	no	No Data	No Data	Deleterious (0)	Probably damaging (1)
CASQ2	1163A > G	E388G	yes	yes	no	yes	No Data	No Data	Tolerated (0.65)	Unknown (0)
SYNE1	17867G > A	R5956H	yes	yes	no	yes	rs80265744	0.04137	Deleterious (0.01)	Probably damaging (0.984)
TBX20	991A > G	T331A	yes	yes	no	yes	rs1420582400	0.0008023	Tolerated (1)	Benign (0)

^a^ Allele frequency were data from gnomAD exomes r2.1.1.

Because there was no result suggesting autosomal recessive inheritance of a certain gene, oligogenic or multifactorial inheritance was considered the genetic mechanism for CHD in the family. The siblings inherited heterozygous variants of different genes from their parents (one from father and another from mother), and the variants may not cause CHD alone, but they may cause CHD when combined. With reference to past reports (see Discussion), we hypothesized that the combination of the *NODAL* variant derived from the father and the *TBX20* variants derived from the mother may be related to the onset of CHD, and we therefore focused on these two variants.

### 3.3. *In silico* analyses suggest *NODAL* and *TBX20* to be candidate genes

**Figure 2:**
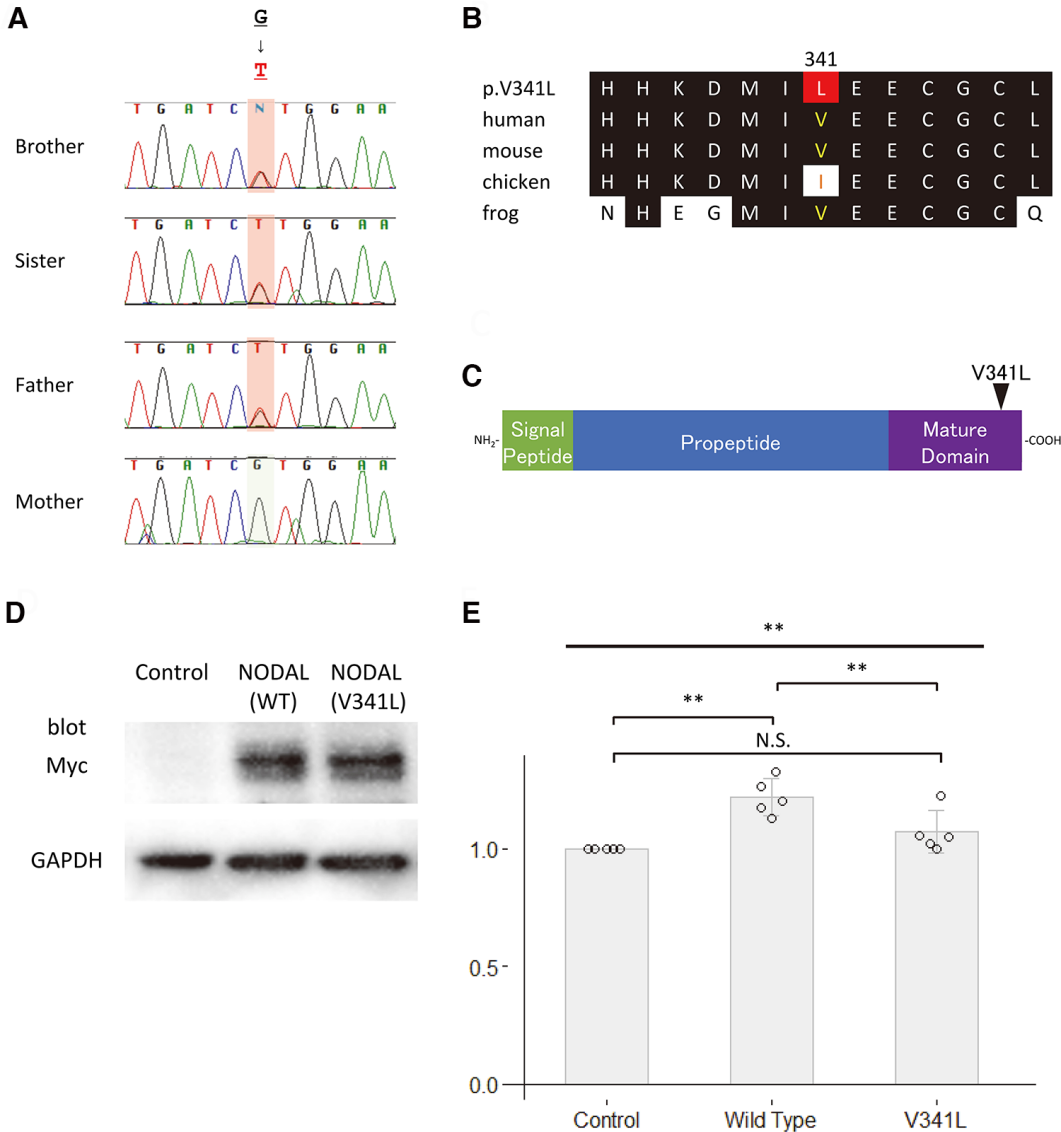
Functional analyses of *NODAL*. (**A**) Sequence chromatograms of *NODAL* in the family. (**B**) The conservation of alignment of the variant in the coding regions between species. Changes in amino acids are highlighted in red. The position of V341 is well-conserved between species. (**C**) Structure of the human NODAL protein. The position of the variant is indicated by an arrowhead. (**D**) Western blot analysis of the c-Myc-tagged wildtype NODAL and the variant NODAL (p.V341l) proteins. Glyceraldehyde-3-phosphate dehydrogenase, GAPDH, is used as an internal control. (**E**) Fold increase in relative luciferase activity in P19 cells transfected with green fluorescent protein (GFP) as control, wildtype *NODAL* or variant *NODAL* (p.V341l) expression construct, and p(SBE)_4_-Luc (*N* = 5). All values are expressed as the mean ± SD. Significance was assessed using one-way ANOVA, followed by Tukey's *post hoc* test. **, *p *< 0.01; N.S., not significant.

**Figure 3:**
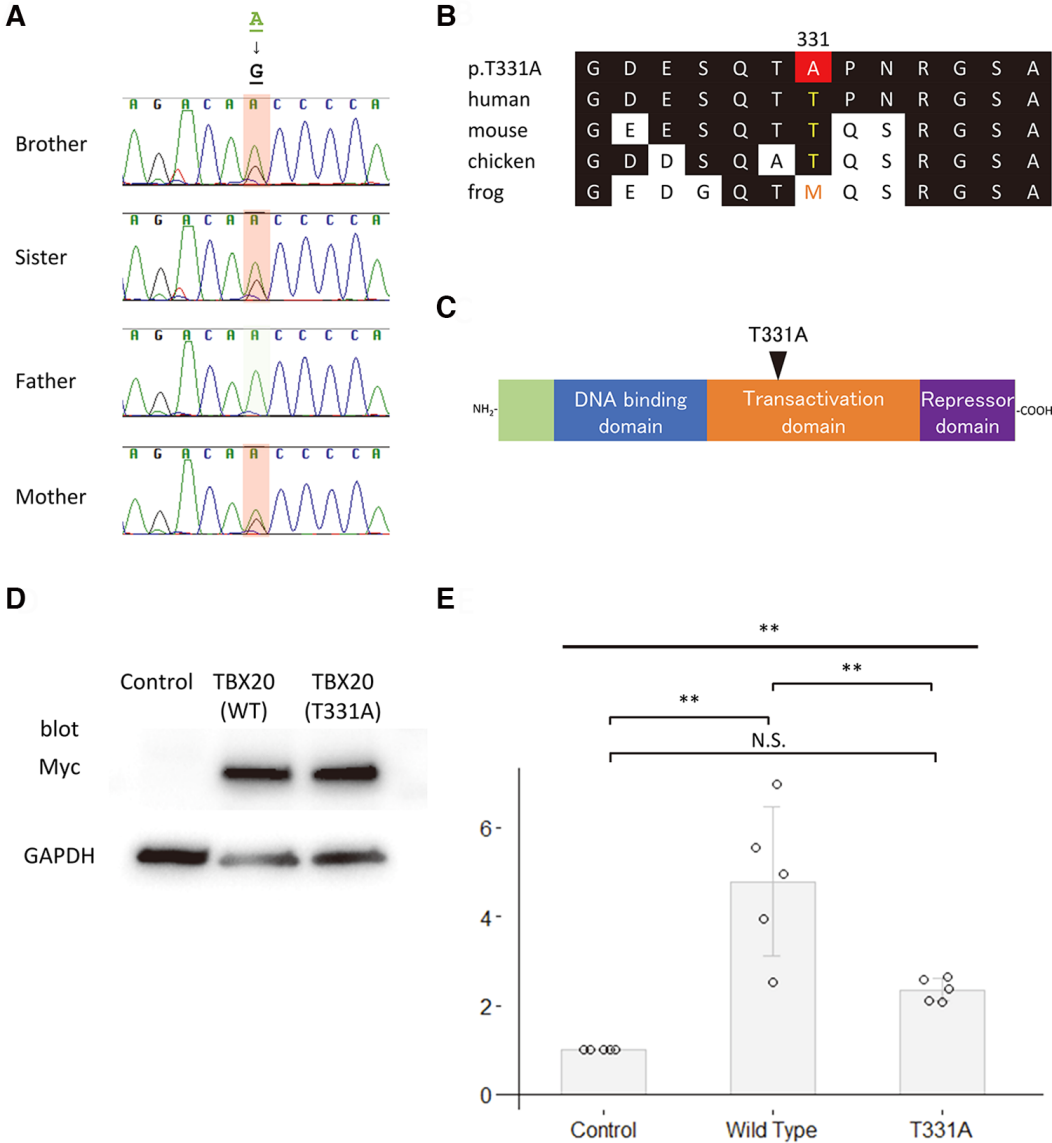
Functional analyses of *TBX20*. (**A**) Sequence chromatograms of *TBX20* in the family. (**B**) The conservation of alignment of the variant in the coding regions between species. Changes in amino acids are highlighted in red. The position of T331 is well-conserved between species. (**C**) Structure of the human TBX20 protein. The position of the variant is indicated by an arrowhead. (**D**) Western blot analysis of the c-Myc-tagged wildtype TBX20 and the variant TBX20 (p.T331A) proteins. Glyceraldehyde-3-phosphate dehydrogenase, GAPDH, is used as an internal control. (**E**) Fold increase in relative luciferase activity in HeLa cells transfected with green fluorescent protein as control, wildtype *TBX20* or variant *TBX20* (p.T331A) expression construct, and *NPPA*-Luc (*N* = 5). All values are expressed as the mean ± SD. Significance was assessed using one-way ANOVA, followed by Tukey's *post hoc* test. **, *p *< 0.01; N.S., not significant.

We performed *in silico* analyses of the variants (Figures 2A–C, 3A–C, and Table 1). The amino acid change p.V341l in NODAL was located in the mature domain, and variants near c.1021G have been reported in patients with various cardiovascular malformations (10). SIFT and PolyPhen-2 analyses predicted that p.V341l was implicated in change of function. The amino acid change p.T331A in TBX20 was located in the transactivation/repression domain (11). A previous study has reported the same variant in a Japanese patient with atrial septum defect (12), and variants near c.991A have been also reported in patients with CHD (13). Therefore, the variants of *NODAL* and *TBX20* identified in this study were likely to be disease-causing.

### 3.4. Both NODAL p.V341l and TBX20 p.T331a fail their function

To assess the impact of the sequence variants p.V341l on the function of NODAL and p.T331A on the function of TBX20, site-directed mutagenesis was performed on human *NODAL* and *TBX20* cDNAs cloned in c-Myc-tagged expression vectors, respectively. Western blot analyses showed that the sizes of both variant proteins of the NODAL p.V341l and the TBX20 p.T331A were same as those of the wildtype proteins (Figures 2D, 3D). Because NODAL, a member of TGF-ß superfamily, activates TGF-ß-regulated SMADs with the cofactors, CRIPTO, CRYPTIC and GDF1, and TBX20, one of the T-box transcription factors, interacts with NKX2.5, GATA4, and GATA5, collaborating to synergistically activate cardiac gene expression, we performed luciferase assay to investigate the transcription activities of the downstream signal signals, SMAD and NPPA. In the luciferase assay, neither NODAL p.V341l variant protein nor TBX20 p.T331A variant protein was able to activate their respective luciferase reporters, p(SBE)_4_ reporter (14) or *NPPA* promoter-luciferase plasmid (15) (Figures 2E, 3E). These results demonstrate that both sequence variants may lead to a functional disturbance of the proteins and may affect the regulation of their downstream target genes.

### 3.5. Nodal and TBX20 double mutant mice exhibit severe perturbation of cardiogenesis

**Figure 4:**
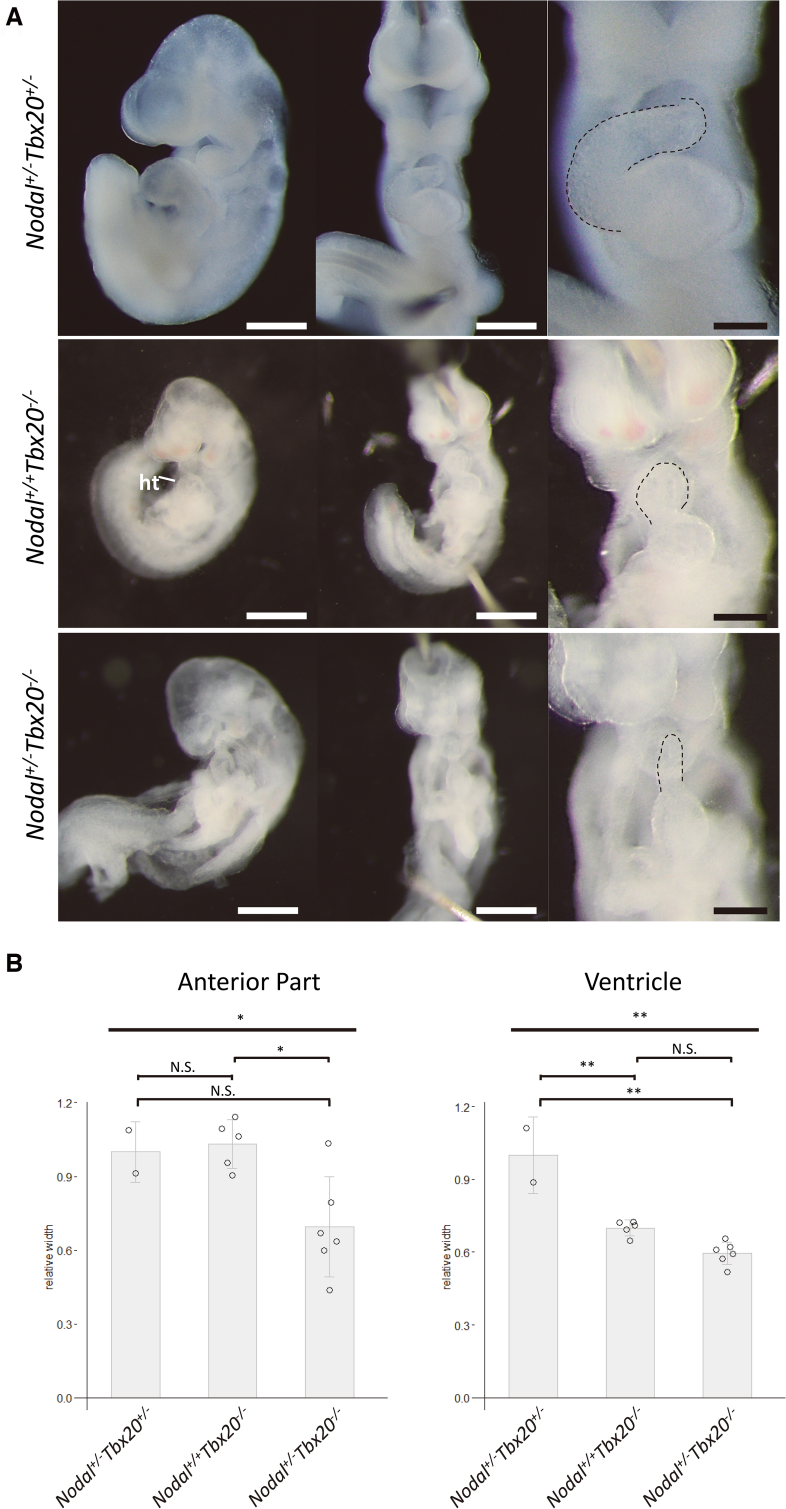
Hypoplasia of anterior part in *NODAL* heterozygous *TBX20* homozygous heart. (**A**) Left lateral views and frontal views of *Nodal^+/+^Tbx20^−/−^* and *Nodal^+/−^Tbx20^−/−^* hearts at embryonic day 9.5. Dotted lines show the anterior parts of the heart tubes. ht, heart. Scale bars, 500 µm in left and middle panels and 200 µm in right panels. (**B**) Bar graphs showing the widths of the anterior or the ventricular part of the hearts. The significant decrease in the width of the anterior parts in *Nodal^+/−^Tbx20^−/−^* hearts. All values are expressed as the mean ± SD. Significance was assessed using one-way ANOVA, followed by Tukey's *post hoc* test. *, *p *< 0.05; **, *p *< 0.01; N.S., not significant.

Because the development of CHD in the siblings was considered to be associated with the presence of both *NODAL* and *TBX20* loss-of-function variants, we analyzed the knockout mice of these two genes to assess if any genetic interaction exists. Embryos homozygous null for *Nodal* die before heart formation is initiated (16), and *Tbx20* homozygous null embryos have arrested development at E9.0 and died at E10.5 with failure of heart development (17–19). Therefore, at first, we generated *Nodal* and *Tbx20* double-heterozygous knockout (*Nodal*^+/−^*Tbx20*^+/−^) mice. Most of the double-heterozygous mutants demonstrated normal cardiogenesis and could survive to adulthood. Although the frequency of double-heterozygous mice around postnatal day 10 was a little lower than that when single-heterozygous mutants of the two genes were bred with each other (data not shown), the cardiac phenotypes of the double-heterozygous mutants were normal and comparable to those of single-heterozygous mutants (Figure 4A). Next, we generated *Nodal* hetero- and *Tbx20* homozygous knockout mice (*Nodal*^+/−^*Tbx20*^−/−^) and compared their cardiac phenotypes with those of *Tbx20* homozygous knockout mice (*Nodal*^+/+^*Tbx20*^−/−^). Although both mutants exhibited delay of embryogenesis with disturbance of cardiac looping, *Nodal*^+/−^*Tbx20*^−/−^ heart showed more severe hypoplasia of the developing heart tube, especially in the anterior part that gives rise to the outflow tract and right ventricular primordium at E9.5 (dotted line in Figure 4A), whereas the ventricle portions were comparable in both genotypes. The width of the anterior part of developing heart tube was significantly narrower than that in *Nodal*^+/−^*Tbx20*^−/−^ (Figure 4B), suggesting a genetic interaction between *Nodal* and *Tbx20* during early heart development.

**Figure 5:**
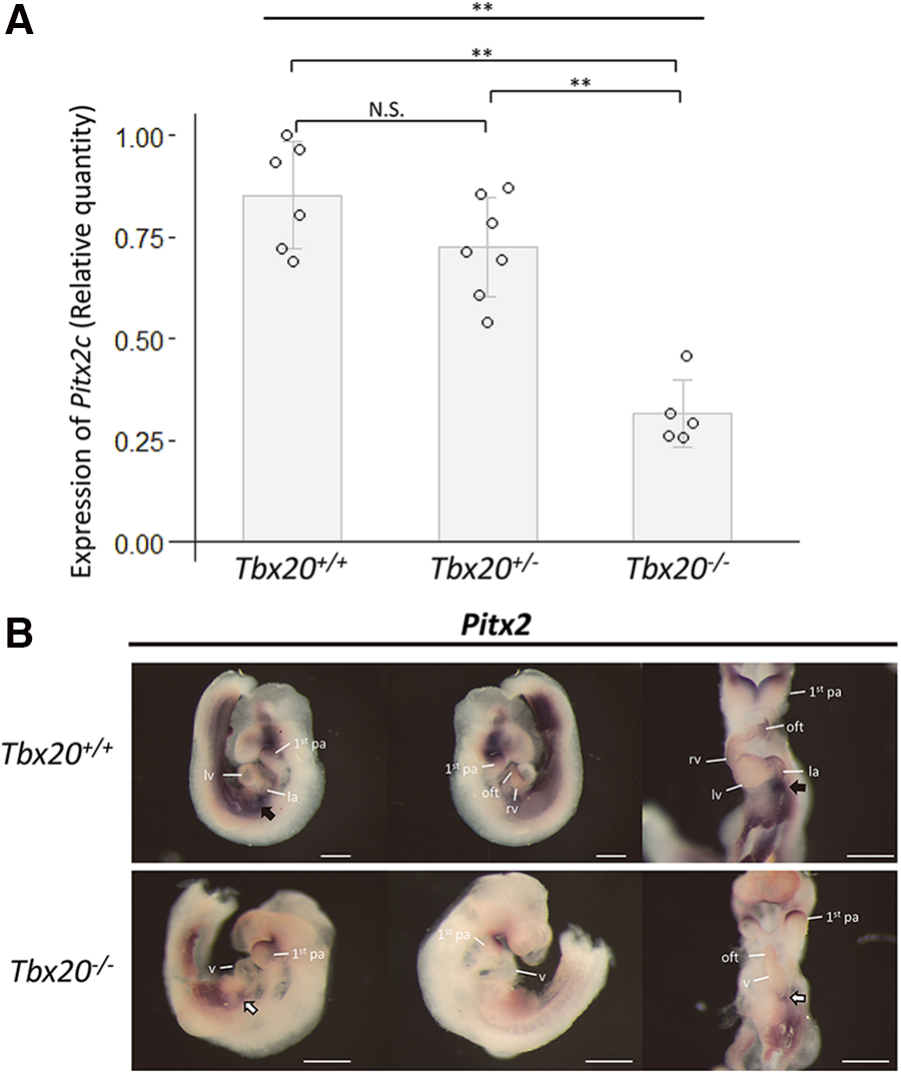
Decreased expression of Pitx2 in *TBX20* homozygous embryos. (**A**) Quantitative RT-PCR results show the significant decrease in *Pitx2c* expression in *Tbx20^−/−^* embryos, compared with that in *Tbx20^+/+^* or *Tbx20^+/−^* at embryonic day 9.5. *Gapdh* was used as internal control. Data were analyzed using one-way ANOVA, followed by Tukey's *post hoc* test. **, *p *< 0.01; N.S., not significant. (**B**) Whole-mount *in situ* hybridization with *Pitx2* antisense riboprobe. *Pitx2* expression dramatically decreases in the area of the cardiac inflows of *Tbx20^−/−^* embryos (white arrows in the lower panels), compared to that of *Tbx20^+/+^* (black arrows in the upper panels) at embryonic day 9.5. 1st pa; first pharyngeal arch; la, left atrium; lv, left ventricle; oft, outflow tract; rv, right ventricle; v, ventricle. Scale bars, 500 µm.

To explore the molecular mechanism underlying the genetic interaction of *Nodal* and *Tbx20*, expression analyses of a candidate downstream molecule, PITX2, was performed using *Tbx20* mutant embryos; PITX2 is a well-known downstream target of NODAL (20–23). Quantitative RT-PCR using primers for *Pitx2c* exhibited significant decrease of *Pitx2c* expression in *Tbx20* homozygous null mutants compared to that in wildtype (*Tbx20^+/+^*) or *Tbx20^+/−^* embryos (Figure 5A). Whole-mount *in situ* hybridization showed a dramatic decrease in the expression of the developing hearts in *Tbx20* null embryos (white arrows in Figure 5B), compared to that in wildtype embryos (black arrows in Figure 5B). These results suggest that PITX2 is a common downstream effector of both NODAL and TBX20.

## 4. Discussion

In this study, we were challenged to elucidate the genetic origin of an intriguing family with two affected siblings, one miscarriage fetus, and both healthy parents. Although we could not analyze the genome of the miscarried fetus, the genomes of the parents and both siblings were subjected to trio-based gene panel analysis by NGS, resulting in the identification of two heterozygous variants in *NODAL* (c.1021G > T, p.V341l) and *TBX20* (c.991A > G, p.T331A) common to siblings and to just one of parents. These two variants had a very low allele frequency and were suggested to be pathogenic *in silico*; decreased transcription activities of downstream signaling pathways were observed *in vitro.* Our analyses of genetically engineered *Nodal^+/+^Tbx20^−/−^* and *Nodal^+/−^Tbx20^−/−^* mouse embryos demonstrated a genetic interaction between Nodal and Tbx20 *in vivo* where *Nodal^+/−^Tbx20^−/−^* embryos showed more severe defects than did *Nodal^+/+^Tbx20^−/−^* embryos during early heart development. These results suggest that *NODAL* and *TBX20* are complementary for the cardiac development, and a combinatorial loss-of-function of *NODAL* and *TBX20* could be implicated in the etiology of CHD.

The way by which the combinatorial loss-of-function of *NODAL* and *TBX20* can cause complex CHD associated with single ventricular morphology was not fully elucidated in this study. NODAL, a TGF*β*-related signaling molecule, is regulated extracellularly by the epidermal growth factor-Cripto-1/FRL-1/Cryptic (EGF-CFC) cofactors and antagonists of the Lefty and Cerberus families of proteins, controlling mesoderm and endoderm formation, the positioning of the anterior-posterior axis, and left-right axis specification (24). Mice homozygous null for *Nodal* lack a primitive streak, displaying only sporadic formation of some posterior mesoderm (16, 25); the heterozygous mutant has no phenotype. Human genetic studies have shown that some variants of *NODAL* cause heterotaxy syndrome, which is often associated with single ventricular morphology, with incomplete penetrance (10, 26–28). A recent study demonstrated that Nodal regulates asymmetric morphogenesis of the embryonic heart tube by generating helical shape (29). By contrast, Tbx20, a T-box transcription factor, is expressed in cardiomyocytes throughout heart development, and mice homozygous null for *Tbx20* experienced arrested development at E9.5 with hypoplastic unlooped hearts (Figure 4A; *Nodal^+/+^Tbx20^−/−^*), in part due to decreased cardiomyocyte proliferation (17–19). Numerous variants of *TBX20* have been reported to be associated with various CHDs, including hypoplastic right heart syndrome, a type of single ventricular morphology, as well as cardiomyopathy (15, 30–34). Although we failed to show any complex CHD in *Nodal^+/−^Tbx20^+/−^* mutant mice, *Nodal^+/−^Tbx20^−/−^*mutants resulted in severe hypoplasia of ventricular primordium and probably defects of early heart looping (Figure 4). Taken together, we speculate that the combinatorial loss-of-function of *NODAL* and *TBX20* could cause hypoplasia of one ventricle and the looping defect, resulting in single ventricular morphology with or without heterotaxy. Why the detailed phenotypes differ between siblings with single ventricle is still obscure, although we speculate other unidentified modifier gene(s), epigenetic factor(s), stochastic event(s), and/or environmental factor(s) might be involved.

In this study, *Pitx2* expression was downregulated in *Tbx20* null embryos. A previous report suggested that PITX2 might be a direct target of TBX20, using chromatin immunoprecipitation with high throughput sequencing data (35). PITX2 is a downstream target molecule of NODAL (36). Therefore, PITX2 is likely to be a common downstream target of NODAL and TBX20 during the cardiac development. *Pitx2* generates three isoforms, *Pitx2a, b*, and *c*, and *Pitx2c* is mainly expressed in the heart, driving the asymmetrical cardiac morphogenesis (20, 22, 23). *Pitx2* knockout mice die before birth and demonstrate right isomerism with double outlet right ventricle, reminiscent of our patient with heterotaxy, and atrial/ventricular septal defects. These findings molecularly suggest that downregulation of *PITX2* expression caused by an additive or synergistic effect of the combinatorial loss-of-function of *NODAL* and *TBX20* during early heart development, probably the looping stage, may result in single ventricular morphology and heterotaxy.

Other than the variants in *NODAL* and *TBX20*, we also detected variants in the two genes *CASQ2* and *SYNE1*, common to the siblings. Calsequestrin 2 encoded by *CASQ2* is localized in the sarcoplasmic reticulum in cardiomyocytes, functioning as a Ca^2+^ binding protein that stores Ca^2+^ for cardiomyocyte contraction (37). Mutations in *CASQ2* cause fatal ventricular arrythmia, referred to as catecholaminergic polymorphic ventricular tachycardia 2 (CPVT2) (38, 39), and the C-terminal end of this gene, where the variant (E388G) we found is located, is known to be functionally important (40). However, they have not been implicated in CHD. *SYNE1* encodes a spectrin repeat-containing nuclear envelope, localized to the nuclear membrane in skeletal, smooth, and cardiac muscle cells (41, 42). *Syne1*-knockout mice exhibited decreased survival rates, growth retardation, and a significantly lower exercise capacity due to nuclear dysfunction in skeletal muscle, but no cardiac phenotype (43). Mutations in *SYNE1* cause neuromuscular diseases, including spinocerebellar ataxia 8 (44, 45), Emery-Dreifuss muscular dystrophy 4 (46), and arthrogryposis multiplex congenita 3 (47), and there is a report that the variants on *SYNE1* are involved in skeletal muscle disorder and cardiomyopathy (46). It may be possible that variants of *SYNE1* can be associated with CHD as *MYH7*, a previously known to be a cardiomyopathy causing gene, is recently reported to be associated with CHD (48). Given these studies, it is a limitation of this study that we cannot completely rule out the effects of these *CASQ2* and *SYNE1* variants as well as the variants of genes that were not included in our gene analysis panel. They might be genetic modifiers for the phenotypes of siblings in our family, although their contributions are considered to be weak.

In conclusion, *via* a combination of NGS and *in vitro* and *in vivo* analyses, we show possible evidence of digenic inheritance in a family with complex CHD. Such an approach may provide insights into the pathogenesis of CHD with multifactorial nature.

## Data Availability

The data used to support the findings of this study are included in the main text or supplementary material. We submitted the novel NODAL and CASQ2 variant data in ClinVar and GenBank, respectively. Any remaining information is available from the corresponding author upon reasonable request.
